# Approach for Self-Calibrating CO_2_ Measurements with Linear Membrane-Based Gas Sensors

**DOI:** 10.3390/s16111930

**Published:** 2016-11-17

**Authors:** Detlef Lazik, Pramit Sood

**Affiliations:** Helmholtz Centre for Environmental Research—UFZ, Theodor-Lieser-Strasse 4, Halle (Saale) D-06120, Germany; pramit.sood@ufz.de

**Keywords:** gas sensors, membrane, internal standard, monitoring, greenhouse gases, CO_2_

## Abstract

Linear membrane-based gas sensors that can be advantageously applied for the measurement of a single gas component in large heterogeneous systems, e.g., for representative determination of CO_2_ in the subsurface, can be designed depending on the properties of the observation object. A resulting disadvantage is that the permeation-based sensor response depends on operating conditions, the individual site-adapted sensor geometry, the membrane material, and the target gas component. Therefore, calibration is needed, especially of the slope, which could change over several orders of magnitude. A calibration-free approach based on an internal gas standard is developed to overcome the multi-criterial slope dependency. This results in a normalization of sensor response and enables the sensor to assess the significance of measurement. The approach was proofed on the example of CO_2_ analysis in dry air with tubular PDMS membranes for various CO_2_ concentrations of an internal standard. Negligible temperature dependency was found within an 18 K range. The transformation behavior of the measurement signal and the influence of concentration variations of the internal standard on the measurement signal were shown. Offsets that were adjusted based on the stated theory for the given measurement conditions and material data from the literature were in agreement with the experimentally determined offsets. A measurement comparison with an NDIR reference sensor shows an unexpectedly low bias (<1%) of the non-calibrated sensor response, and comparable statistical uncertainty.

## 1. Introduction

Several gas measurement technologies currently exist within e.g., science, medicine and industry, such as solid-state gas sensors (solid electrolyte, chemo-resistive and calorimetric); sensors based on polymers or Carbon Nano Tubes (CNTs), optical gas sensors based on spectroscopic or specific wavelength absorbance techniques, and conventional gas chromatography. These sensor technologies, covering a wide range of application areas such as manufacturing and production, the automotive industry, indoor air quality control, medical diagnostics and environmental monitoring, have been reviewed, e.g., in [1,2,3,4].

Gas sensors based upon optical principles [3,5] and zeolite materials [6] have been shown to exhibit generally high selectivity or to enable the selective estimation of a particular gas component in a gaseous matrix, precisely. Methods such as gas chromatography, different spectroscopic measurement techniques, acoustic sensing [7], the integration of several potential measurement methods into a sensor array/electronic nose [8,9,10] and the more recently used technology of pattern recognition [11], can be applied for multi-component analysis of a gaseous mixture. The applicability of a particular measurement technique depends on temperature, pressure, expected concentration range, phase constitution and heterogeneity of the observation object, its chemical composition and the existence of explosive, aggressive or sensor-poisoning substances. Table 1 shows CO_2_ measurement techniques that have been discussed with regard to application parameters, advantages, and disadvantages.

The techniques in Table 1 are applicable mainly for local measurement in/of gaseous phases; phase separation is needed for measurement in liquids [23,24,25,26]. An alternative measurement technique applicable for representative CO_2_ measurement in mixed liquid and gaseous phases with spatially varying CO_2_ content first demonstrated in [27] is based on the selective permeation of gases through membranes into hollow measurement chambers.

Before a measurement step, such a measurement chamber will be flushed with a purge gas to adjust reproducible steady-state flow conditions (dynamic equilibrium) for the ambient gas components within the gas selective membrane. During the consecutive measurement step, a characteristic change of mole numbers is caused within the measurement chamber near the adjusted steady-state gas flows. This change can be monitored either in terms of a volumetric change or a pressure change, and can be used to determine individual gas concentration, e.g., combining different selective measurement cells (combinations of membrane-coated chamber and a gas-tight reference chamber) [27,28]. Such membrane-based gas sensors could be advantageous in measurement tasks where only a single gas component changes. Instead of the need to use different gas-selective membranes for this case, only a single gas-selective membrane, i.e., only a single measurement cell is needed.

The shape of such a membrane-based gas sensor can be designed based on the properties of the object of observation, which brings advantages as well as disadvantages. Besides providing a local measurement, application of tubular membranes enables a representative measurement in large, heterogeneously composed environments, such as soils, aquifers, disposal sites, reactors etc., and can replace here a high number of necessary sensors with local detection. Depending on the membrane properties, such a sensor can be exposed to explosive, aggressive or poisoning substances and applied for different temperatures and pressures in different fluid phases. A resulting disadvantage, however, is that the sensor must be calibrated. Its response depends, e.g., on the operating conditions, the sensor geometry, membrane material and the target gas component. For instance, during installation of linear gas sensor tubes (called line-sensors) in a soil, site-specific individual properties such as different line-sensor lengths can be, in practice, a consequence. Therefore, individual response characteristics result. These characteristics and their temporal development have to be understood for each installed line-sensor and considered in terms of individual (time-dependent) sensor slopes. The aim of this study is to give an alternative to such a multi-criteria slope dependency. To normalize the sensor response, an approach is developed for single gas component measurements based on an internal gas standard. As an example, different mixtures of CO_2_ and dry air (referred to as air) will be considered.

## 2. Theoretical Description

As shown in [27], the steady-state diffusive flows (dynamic equilibrium) of gas molecules between an outer (index “*a*”) and an inner face (index “*i*”) of a gas-selective membrane result in a pressure change $a_{1}$ (mbar/s):

(1) $$\frac{dp^{i}}{dt}=a_{1}=p^{a}gP_{x}\sum_{j}f_{jx}(\chi_{j}^{a}-\beta \chi_{j}^{i})$$

within a measurement chamber, covered with that membrane. Considering a linear gas sensor with a tubular membrane, the geometric factor *g* (m^−2^) in Equation (1) is $g=2\pi \cdot L/(V_{0}\ln(R_{a}/R_{i}))$, (L (m)—length of tube, $V_{0}$ (m^3^)—gas volume in the tube, $R_{a},R_{i}$ (m)—outer and inner radius of tube), $P_{x}$ (m^2^/s) is the permeability of gas component “*x*” in the membrane, $f_{jx}=P_{j}/P_{x}$ is the permeation selectivity (referred to as selectivity) of gas component “*j*” with respect to gas component “*x*”, the ratio of gas pressures inside and outside the tube is described by $\beta =p^{i}/p^{a}$, and $\chi_{j}^{i},\;\chi_{j}^{a}$ are the mole fractions of a gas component “*j*” within the chamber and outside. Equation (1) forms the basis for a multi gas component analysis [27] as well as for analyzing a single gas component. Considering a mixing process of a gas component with a gaseous matrix $\left\{\chi_{1},\chi_{2}\ldots \right\}$ (referred to as ${\left\{\chi_{j}\right\}}_{j=1,2\ldots }$), such single gas component analyses are demonstrated for O_2_ and CO_2_ using a single gas-selective membrane [28].

**Figure 1:**
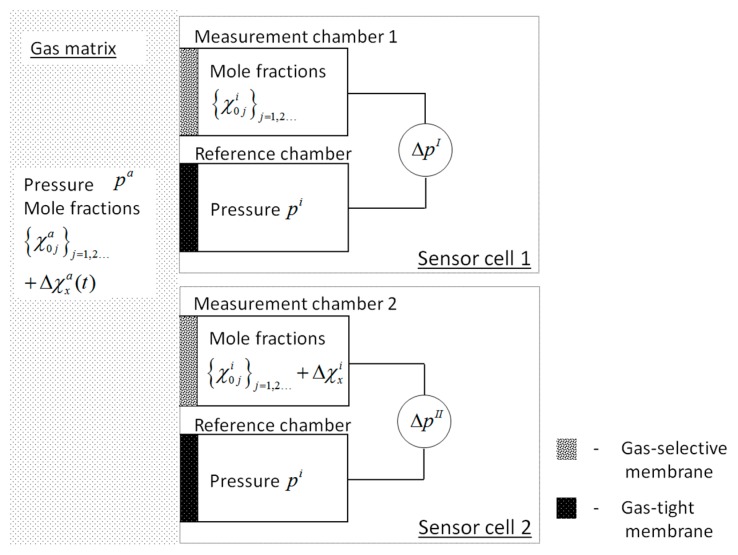
Combined sensor cell: The quantity $\Delta \chi_{x}^{i}$ in measurement chamber 2 serves as an internal standard for a calibration-free determination of $\Delta \chi_{x}^{a}$.

In an open system e.g., air within a facility, outdoors or in unsaturated soil, a local addition of a gas component causes the dilution of ambient gas components, resulting in locally changing mole fractions. Assuming the initial mole fractions ${\left\{\chi_{j}(t_{0})\right\}}_{j}={\left\{\chi_{0j}\right\}}_{j}$ of a gas phase are known at time $t_{0}$ and the mole fraction $\Delta \chi_{x}$ is added at time $t>t_{0}$, the gas composition changes to ${\left\{\chi_{j}\right\}}_{j}$:

(2) $${\left\{\chi_{j}\right\}}_{j=1,2\ldots }={\left\{\delta_{jx}\Delta \chi_{x}+\left(1-\Delta \chi_{x}\right)\cdot \chi_{0j}\right\}}_{j=1,2\ldots }$$

($\delta_{jx}$ is the Kronecker delta). The response $a_{1}^{I}$ of a membrane-based gas sensor according to sensor cell 1 in Figure 1 to this changed gas composition, calculated from the resulting pressure evolution $\Delta p^{I}(t)$ near the dynamic equilibrium can be found with respect to Equation (1):

(3) $$a_{1}^{I}=\frac{\Delta \chi_{x}^{a}}{k^{a}}+a_{1s},\;k^{a}=\frac{\tau_{x}}{p^{a}(1-\sum_{j}f_{jx}\chi_{0j}^{a})},\;a_{1s}=\frac{p^{a}}{\tau_{x}}\sum_{j}f_{jx}\;(\chi_{0j}^{a}-\beta \;\chi_{0j}^{i}).$$

Here $\tau_{x}={\left(gP_{x}\right)}^{-1}$ is a gas component-specific response time constant of the measurement cell. The slope $k^{a}$ in Equation (3) called hereafter the “outer slope” specifies the unknown mole fraction change $\Delta \chi_{x}^{a}$ with respect to a reduced (dynamic) pressure change $a_{1d}=a_{1}^{I}-a_{1s}$. The offset $a_{1s}$ considers the contribution of the initial gas compositions at both membrane faces to the measurement signal.

The outer slope and the offset have to be calibrated in Equation (3). Alternatively, an internal standard as an on-line reference signal can be used. To this end, a sensor set-up is designed according to Figure 1, combining two sensor cells. Each sensor cell is made from a measurement chamber coated with a gas-selective membrane (grey) and a geometrically equal reference chamber coated with a gas-tight membrane (black). For the internal standard, the quantity $\Delta \chi_{x}^{i}$ of the target gas component will be added to the purge gas ${\left\{\chi_{0j}^{i}\right\}}_{j}$ of sensor cell 2 in Figure 1, forming a pressure change $a_{1}^{II}$ that can be calculated near the dynamic equilibrium from the pressure evolution $\Delta p^{II}(t)$. Applying Equation (2) to Equation (1) and considering (1) measurement cells of the same construction, which (2) are exposed to the same outer gas matrix ${\left\{\chi_{j}^{a}\right\}}_{j}$, (3) where the gas composition ${\left\{\chi_{0j}^{i}\right\}}_{j}$ within measurement chamber 1 should be equal to the initial gas composition in measurement chamber 2, the pressure change $a_{1}^{II}$ is:

(4) $$a_{1}^{II}=a_{1}^{I}-\frac{\Delta \chi_{x}^{i}}{k^{i}},\;k^{i}=\frac{\tau_{x}}{p^{i}(1-\sum_{j}f_{jx}\chi_{0j}^{i})}$$

where $k^{i}$ forms the “inner slope” of sensor response with respect to the applied internal standard.

Combining the concentration-dependent signal dynamics $a_{1d}$ from Equation (3) with Equation (4), and considering the initial mole fraction $\chi_{0x}^{i}$ within the measurement chambers, results in:

(5) $$\Delta \chi_{x}^{a}=\frac{k^{a}}{k^{i}}\alpha \cdot \Delta \chi_{x}^{i},\;\alpha =\frac{a_{1d}}{a_{1}^{I}-a_{1}^{II}},\;a_{1d}=a_{1}^{I}-a_{1s}$$

and the total mole fraction $\chi_{x}^{a}=\chi_{0x}^{a}+(1-\chi_{0x}^{a})\Delta \chi_{x}^{a}$ will be obtained with respect to Equation (2).

The ratio $k^{a}/k^{i}$ contains known properties, i.e., the selectivities of the membrane material and the initial gas compositions at both membrane faces. Considering, e.g., similar initial mole fractions of outer gas matrix and purging gas, i.e., the case where the monitored air within a facility or outdoors is also used as the initial gas for purging the sensor chambers, the slope ratio converges against $k^{a}/k^{i}\to p^{i}/p^{a}=\beta$. In this case, the partial pressure of gas component *x* is determined by the initial mole fraction $\chi_{0x}^{i}$, the mole fraction $\chi_{x}^{i}$ adjusted in cell 2 and the scaled pressure change α. The outer mole fraction follows with

(6) $$\chi_{x}^{a}=\beta \;\alpha (\chi_{x}^{i}-\chi_{0x}^{i})+\chi_{0x}^{i}$$

where the offset

(7) $$a_{1s}=\frac{p^{a}-p^{i}}{\tau_{x}}\sum_{j}f_{jx}\;\chi_{0j}^{i}$$

depends now only on the initial pressure difference at both faces of the membrane.

## 3. Experimental Proof

### 3.1. Setup and Experimental Realization

Two line-sensors were prepared combining tubular gas selective PDMS membranes (Versilic SPX-50 tubing, Saint Gobain Performance Plastics Corporation, Farmington Hills, MI, USA) with tubular gas-tight reference membranes of the same size. For preparation of these reference membranes, a significantly less permeable C-Flex tubing [27] (Saint Gobain Performance Plastics Corporation) was used. The tubes (2*R_i_* = 1/32″, 2*R_a_* = 3/32″ were cut into pieces of equal length *L* = 2.47 m. Approximate values of permeability coefficient of the Versilic SPX-50 membrane are provided by the supplier as $\{P_{CO2},\;P_{O2},\;P_{N2}\}=\{3525,\;661,\;320\}\times 10^{-10}$ cm^3^cm/cm^2^/s/cmHg [29].

**Figure 2:**
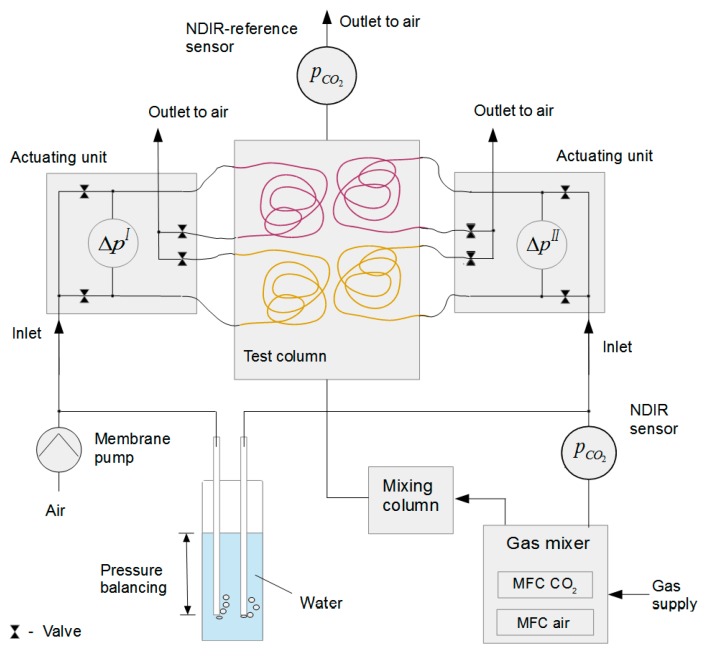
Sketch of the experimental setup. Selective membrane tubes (**red**) and gas-tight reference tubes (**yellow**) are placed in the test column that is flushed by different mixtures of CO_2_ and air.

The experimental setup that was used to test the new approach is shown in Figure 2. The prepared line-sensors were inserted into a column (*V* = 1.7 L), referred to as the test column, which was flushed from bottom to top with a gas mixture composed of air and CO_2_ of varying content. The cyclic membrane-based gas measurement was started with conditioning of a line-sensor to adjust steady state fluxes through the membranes between the gas components in the test column and the purge gas within the line-sensor tubes. Line-sensor 1 was purged by dry air using a membrane pump. Mass flow controllers (MFC 8712, Bürkert Fluid Control Systems, Ingelfingen, Germany) were used for the flushing of line-sensor 2 with defined mixtures of dry air and CO_2_. After this conditioning step, the line-sensor tubes were closed at both ends for a measurement step using a pinch valve. During this step, the difference pressure between the corresponding measurement/reference chambers was observed by a differential pressure sensor (pressure range ±12.5 mbar, HCLA12X5DB, Sensortechnics, Puchheim, Germany). Consecutively, the pinch valve was opened again for the next conditioning step. The time span for conditioning was set at 30 s, and a time span of 5 s was used for a measurement step. An offset time of 0.5 s was adjusted between closing a measurement chamber and recording of the pressure. 

The line-sensors were run sequentially one after another, i.e., while the pressure evolution within line-sensor 1 is being recorded, conditioning of line-sensor 2 is performed. For a comparison of simultaneous line-sensor signals, the readings in each record were linearly interpolated to each other. The time-delayed response of the NDIR reference that was placed outside the experimental column in the gas outlet was shifted back in time based on a cross-correlation with the signals of line-sensors for direct comparability of the individual measurement values. The individual time-lags shown in Table A1 (Appendix A) correspond to a shift by 20 ± 3 measurement points.

Actuating units containing a pinch valve, a pressure sensor, and electronic control elements were placed near the test column and connected to the sensor membranes by C-Flex tubing. Application-specific firmware was developed to carry out the cyclic measurement procedure, data acquisition, and computation of $a_{1}^{I}$ and $a_{1}^{II}$.

An NDIR CO_2_ probe (CarboCap, GMP-221, range 0–5%_vol_, VAISALA, Helsinki, Finland) was installed between the MFCs and line-sensor 2 to monitor the concentration of the internal CO_2_ standard. The purge gas overpressures for both line-sensors were kept adjusted to about 11 mbar throughout all experiments using glass tubes that were dipped into a water-filled vessel. Therefore, assuming a linear pressure drop, the mean purge gas overpressure within the line-sensors will be close to 5.5 mbar.

The test column gas mixtures were provided by two MFCs (MFC8710, Bürkert Fluid Control Systems; ranges: 0–0.25 L/min for CO_2_ and 0–5 L/min for air). To improve gas homogeneity, the mixtures were flushed through a mixing column (*V* = 1.7 L) first and from there in the test column. At the outlet of the test column, an NDIR CO_2_ probe (CarboCap, GMP-221, range 0–10%_vol_, VAISALA) was installed as an independent reference using a short wide tube. From there, the test gases escaped to the atmosphere. For the adjusted flow rate of 1.5 L/min (Table 2), the pressure build-up within the test column was negligible (checked using a tube connected to the column and dipped into a water-filled open vessel).

### 3.2. Mixed Gas Experiments

Cascaded air-CO_2_ mixtures were flushed with a constant flow rate through the test column within a range of up to 7%_vol_ CO_2_. The CO_2_ concentrations of the internal standard and within the test column were recorded by the NDIR sensors. The temperatures of the purge gases and gases in the test column were monitored. The purge gas for line-sensor 2 was provided from defined air + CO_2_ mixtures with different CO_2_ concentrations. Experimental details are given in Table 2 and Table A1 (Appendix A).

Experiments 1–3 that were performed with different CO_2_ concentrations of the internal standard will be used for a stepwise evaluation of the proposed new approach. Experiment 4 that was carried out for an expanded range of temperature will be used for a discussion of the influence of temperature on the sensor response.

## 4. Results and Discussion

### 4.1. Normalization of Sensor Response

The linearity between $\Delta \chi_{x}^{a}$ and $a_{1}^{I}$ in Equation (3) has already been experimentally demonstrated for mixing oxygen and nitrogen within a large mole fraction range of $\chi_{O2}^{a}=0.01-1$ [28] and for CO_2_ mixed with air for a range $\chi_{CO2}^{a}\leq \;1$ [30] for laboratory conditions. Within these linearity ranges, such a membrane-based sensor can be calibrated by a gas-specific linear characteristic. To that end, the responses $a_{1}^{I}$ have to be compared with reference gases of known composition [27,28,31,32].

However, the outer slope $k^{a}$ depends strongly on the time constant $\tau_{x}={\left(g\;P_{x}\right)}^{-1}$ formed by the geometric factor *g* and the permeability $P_{x}$ of the target gas component. Insofar as different sensor arrangements cause different slopes, also the replacement of a selective membrane by a membrane of the same chemical formulation and geometry could cause a change in slope, e.g., due to changes in manufacturing. For instance, we report an experimentally determined value of about 21%_vol_ s/mbar for CO_2_ measurement with a tubular PDMS membrane of 10 m length, an inner radius of 0.7 mm and an outer radius of 1.8 mm [31]. In contrast, for a 40 m-long PDMS membrane with an inner radius 2 mm and outer radius of 3.5 mm, we determined a slope of about 190%_vol_ s/mbar for the same range of 0–5%_vol_ CO_2_ that was added to air [32].

Depending on the line-sensor properties (geometry, permeabilities), a sufficient large gas-tight vessel has to be used for a usual calibration and sufficient large equilibration times has to be considered, which in turn could result in a huge demand of calibration gases. Alternative to this direct calibration, the calibration gases can be flushed also through the line-sensor instead of the purge gas (inverse calibration), which enables the calibration of installed line-sensors without their dismounting [31].

In contrast to that single sensor response, the dynamic behavior of the reference-based sensor response α is, according to Equation (5), independent of $\tau_{x}$, which is only included in the offset constant $a_{1s}$ in Equation (3). The ratio $k^{a}/k^{i}$ compensates for this combined line-sensor response for differences between the initial outer and inner gas compositions. Hence, under given measurement conditions, one expects similar responses for sensors of different geometry, i.e., for planar- or tubular-designed sensor cells, different lengths of line-sensor or different membrane thicknesses. Moreover, the dynamic range of such a sensor must be independent of the target gas component. Therefore, an internal standard-based gas sensor that is adjusted for a gas component, e.g., for CO_2_, should answer with the same slope (with permeability/selectivity-dependent physical resolution) to another target gas, e.g., argon, chlorine, or hydrogen, as long as the membrane shows permeability differences for this target gas with respect to the initially present gas components. Finally, the application and combination of different membrane materials will be possible, which are best adapted to the target gas component(s) and environmental properties, without changing the internal standard-based (and therefore normalized) response behavior.

Based on the performed experiments, the next sections consider and prove this normalized response behavior for the measurement of the target gas CO_2_ in air.

### 4.2. Preparation of Datasets

**Figure 3:**
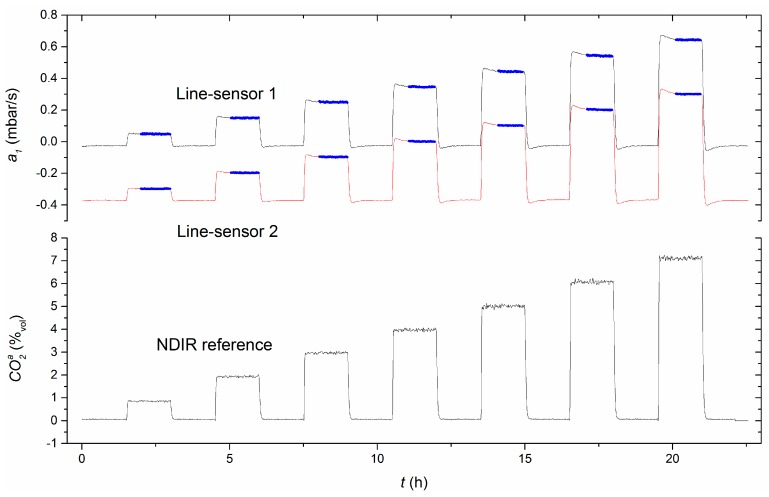
Line-sensor signals for different test column concentrations of CO_2_ (recorded with the NDIR-reference) in experiment 2. Line-sensor 1 (**gray**) was purged with air; line-sensor 2 (**red**) with 3.6%_vol_ CO_2_ mixed in air. Plateau regions (**blue**) indicate engaged line-sensor responses.

Figure 3 shows measurement results for experiment 2 as an example in which line-sensor 2 was run with 3.6%_vol_ internal standard concentration. As shown in Figure 3, both line-sensors follow the concentration evolution consistently within the test vessel. Due to the added inner CO_2_ concentration, line-sensor 2 responded with a lower signal than line-sensor 1.

Figure 3 shows an overshooting of the line-sensor responses after the sudden concentration changes, whose magnitude depends on the concentration difference. In this region, diffusive readjustment of dynamic equilibrium, which forms the sensor’s working point, has not yet finished. The line-sensors show a settling time of about 30 min in the case of a rapid change of CO_2_ by more than 1%_vol_. With respect to the comparability of the recorded data to that of the IR reference sensor, plateau regions were investigated, such as the blue region marked in Figure 3, which were interactively extracted without outlier discrimination as continuous datasets.

### 4.3. Offset Determination

An offset adjustment is needed to determine the zero-point of the measurement system under the current set of measurement conditions. To adjust the offset constant $a_{1s}$ in Equation (5), a baseline can be experimentally determined from a time record of $a_{1}^{I}$ for equivalent gas compositions at both faces of the membrane (e.g., from the measurement values between 0–1.3 h in Figure 3). Therefore, baseline records of 1 h with about 100 measurement values were analyzed before each experiment. The experimentally determined offsets range between $-0.023>a_{1s,\exp}>-0.026$ mbar/s with a mean of −0.0246 mbar/s.

However, the offset $a_{1s}$ can also be calculated independently of any experiment according to Equation (7) for the respective measurement conditions, i.e., the gas pressures within and outside the line-sensors, and the initial purge gas composition. The initial purge gas composition {CO_2_, Ar + O_2_, N_2_} was assumed to be {0.038, 21.876, 78.084}%_vol_. Due to its similar permeation coefficients [33], the contents of argon and oxygen were combined. With respect to the permeability estimates and geometrical measures from Section 3.1, and the mean purge gas overpressure of 5.5 mbar, the offset results in $a_{1s,calc}=-0.0195$ mbar/s. Using the permeabilities $\{P_{CO2},\;P_{O2},\;P_{N2}\}\;=\{3800,\;800,\;400\}\times 10^{-10}$ cm^3^cm/cm^2^/s/cmHg from [34] estimated at 35 °C, the offset results in $a_{1s,calc}=-0.0241$ mbar/s for same conditions (the unit of permeability that considers the experimental conditions, i.e., the applied pressure in “cmHg”, was converted by 7598 mm^2^/s = 1 cm^3^cm/cm^2^/s/cmHg).

With respect to the approach of a self-calibrating measurement system, only the calculated offset ($a_{1s,calc}=-0.0241$ mbar/s) with permeabilities from this literature, which fits well with the experimentally determined offsets, will be subsequently considered.

### 4.4. Temperature Dependency

The permeation process of a gas through a polymeric membrane depends exponentially on temperature: the so-called Arrhenius law. Already in 1939, this strong temperature dependency was considered for rubber-like polymeric membranes in [35]. The influence of this dependency on the measurement signal of a membrane-based gas sensor was investigated for an experimental temperature range of 297–302 K in [31]. However, negligible dependency was found within that 5 K range. This unexpected result was attributed to the measurement principle, in which the purge gas partly compensates for the temperature dependency.

**Figure 4:**
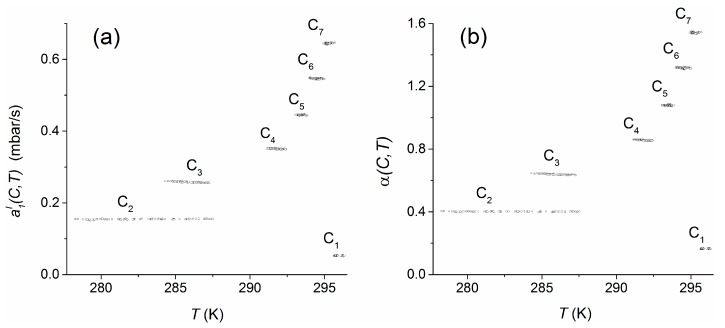
Pressure change $a_{1}^{I}(C,T)$ (**a**) and scaled pressure change α(C,T); (**b**) depending on temperature for the test-column concentrations C_1_–C_7_ (see Table A1, Appendix A) in experiment 4.

Using the data of experiment 4, the temperature dependency was analyzed for an extended range of about 18 K within 278–296 K for the concentration range C_1_–C_7_ (see Appendix A, Table A1) in the test column. The pressure changes α(C,T) using the calculated offset (Section 4.3) and $a_{1}^{I}(C,T)$ comparable to our previous investigations are shown in Figure 4.

Compared to the concentration dependence of the measurement signal, both measures seem to be almost constant within the individual concentration plateaus, i.e., they are not significantly influenced by the temperature. For quantification of the temperature dependencies over the whole temperature range, the regression coefficients $da_{1}^{I}/dT$ and dα/dT, which were obtained by linear regressions of the sensor responses within the individual plateau concentrations (C_1_–C_7_), were weighted with respect to the relative numbers of the plateau-measurement values used. To consider the different widths of temperature ranges that were observes within the individual concentration plateaus, each regression coefficient was, in addition, weighted with the relative temperature range covered by the respective plateau. Finally, the weighted regression coefficients were averaged. As a result, the mean temperature dependencies including standard errors were found to be $\Delta a_{1}^{I}/\Delta T=(-0.37\pm 1.31)\cdot 10^{-4}$ mbar/K and $\Delta \alpha /\Delta T=(-1.84\pm 2.50)\cdot 10^{-4}$ K^−1^. In both cases, the estimated temperature trends do not significantly influence the measures within the applied temperature range.

### 4.5. Scaling Behavior of Combined Line-Sensor Response

Whereas the outer transformation behavior of a combined line-sensor response is defined according to Equation (3) by the ratio $\Delta \chi_{CO2}^{a}/a_{1d}=k^{a}$, the ratio $\Delta \chi_{CO2}^{i}/(a_{1}^{I}-a_{1}^{II})=k^{i}$ in Equation (4) defines the inner transformation behavior of its response. The reciprocal values $1/k^{a}$ and $1/k^{i}$ are the outer and inner sensitivity, respectively, of the combined line-sensor.

According to Section 2 the outer transformation behavior can be calculated in dependence on the measurement conditions for known inner slope. In the present case one held $k^{a}=\beta k^{i}$. However, with respect to the comparison of both signals in Equation (5), the final response behavior of the combined line-sensor signal enables no insight in the internal scaling behavior.

**Figure 5:**
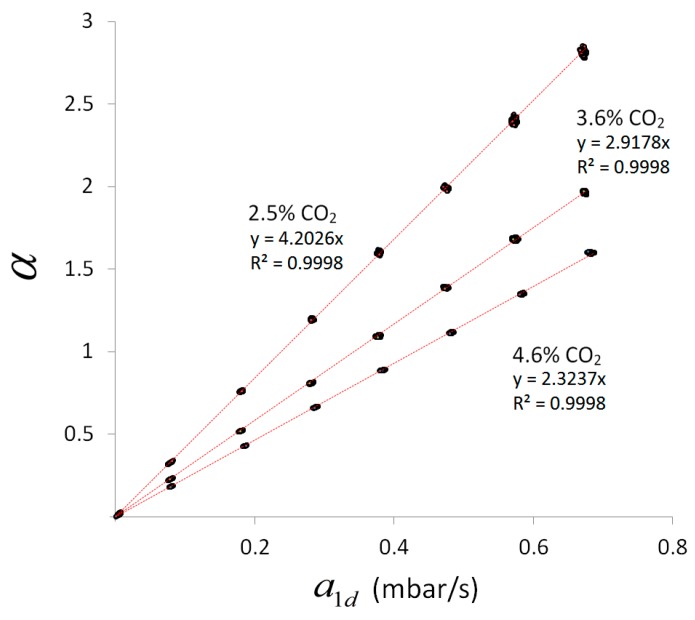
Combined line-sensor **r**esponse α in dependence of the dynamic pressure change $a_{1d}$ compared for various concentrations in the test-column for experiments 1–3. The used internal standard concentrations are indicated near the respective regression lines.

To consider this scaling behavior, Figure 5 shows a comparison of the pure measurement signals: the combined line-sensor response α for experiments 1–3 with respect to $a_{1d}$. The internal standard concentrations used (Table 3) are indicated in Figure 5 near the respective dataset. The measurement values (dots) along a regression line (red) represent the behavior of the combined line-sensor response for a particular internal standard concentration and diverse outer CO_2_ concentration of up to 7%_vol_. Due to the offset subtraction in α and $a_{1d}$, the regression lines have to start near the point of origin.

The regression coefficients $d\alpha /da_{1d}={\left(a_{1}^{I}-a_{1}^{II}\right)}^{-1}$ of the regression lines in Figure 5 increase with decreasing concentration of the internal standard $\Delta \chi_{CO2}^{i}$ (Table 3). This behavior results in inner slopes that are given in Table 3 for the experiments 1–3, and which characterizes the (inner) signal dynamics of the combined line-sensor.

The inner slope can also be calculated according to Equation (4) with respect to the measurement conditions: gas pressures and initial inner gas composition. For given permeabilities and geometrical measures, this theoretical inner slope is 0.033 s/mbar. The most sensitive parameter in Equation (4) that determines this underestimate is the CO_2_ permeability that occurs within time constant $\tau_{CO2}={\left(g\;P_{CO2}\right)}^{-1}$. On the other hand, this permeability is the only parameter that does not change the offset according to Equation (7), behaving comparably to the experimental values (Section 4.3). For a long time [36], the determination of such permeability values has been performed in single gas experiments based on standardized methods, e.g., applying different gas pressures in the range of several bar to both faces of a membrane, analyzing the steady-state gas flow [34] and extrapolating the results to a zero-pressure difference. These material parameters can differ from those for mixed gas systems with comparatively small (partial) pressure differences. A fit based on the experimentally determined mean inner slope ${\bar{k}}^{i}=0.105$ s/mbar results in a 2.5-fold smaller effective CO_2_ permeability of $1.17\times 10^{-9}$ m^2^/s, and a reduced effective selectivity of $f_{CO2,N2}\approx 4$ with respect to N_2_. Such a reduced selectivity has already been observed in previous mixed gas tests, and has been discussed, e.g., in [30]. In addition, the decrease in selectivity can be partly attributed to the non-zero permeabilities of the C-Flex-reference membranes [27].

**Figure 6:**
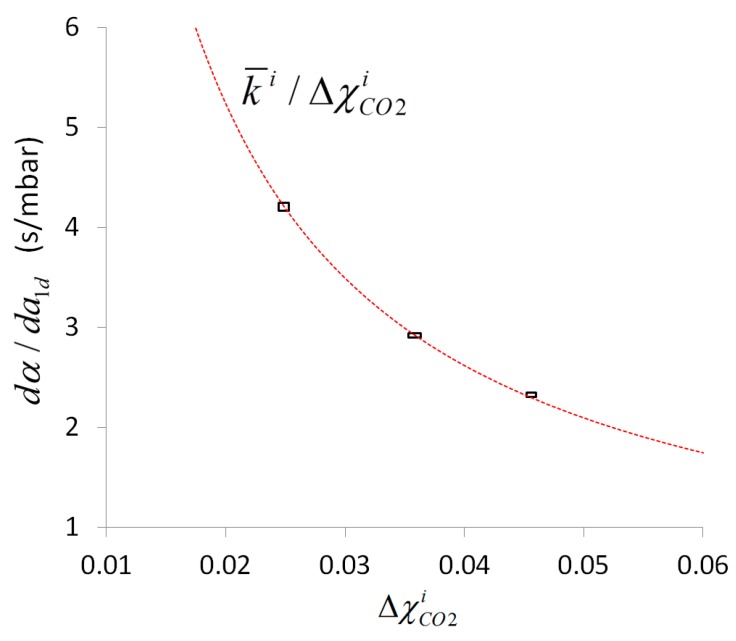
Comparison of calculated ideal behavior ${\bar{k}}^{i}/\Delta \chi_{CO2}^{i}$ (**red** line) and experimentally determined regression coefficients $d\alpha /da_{1d}$. The rectangles are formed by the double-side three-fold standard deviations around the respective means.

Figure 6 shows the determined individual (real) regression coefficients $d\alpha /da_{1d}$ from Table 3 compared to the averaged (ideal) response behavior ${\bar{k}}^{i}/\Delta \chi_{CO2}^{i}$ (red line). The deviation of the inner slopes $k^{i}$ in Table 3 from the mean inner slope is small. For better visibility, the three-fold standard deviations are shown as rectangles around the experimental means for $\Delta \chi_{CO2}^{i}$ and $d\alpha /da_{1d}$. These rectangles match the calculated characteristics, i.e., the sensor signal is highly determined by the ratio ${\bar{k}}^{i}/\Delta \chi_{CO2}^{i}$ and therefore, the inner slope can be considered to a good approximation as independent of the internal standard concentration. Moreover, Figure 6 shows that the same variation of internal standard concentration results for smaller $\Delta \chi_{CO2}^{i}$ in an increased error propagation into the sensor response. Taking both aspects into account, $k^{i}$ is a key parameter that can be calculated based on material parameters and used to configure an application specific optimal design of the combined line-sensor with defined sensitivity (in the present case the inner sensitivity is $1/k^{i}=9.52$ mbar/s and the outer is $1/(\beta k^{i})=9.58$ mbar/s). Moreover, based on the measurement values, this parameter can be recorded, e.g., in terms of a technical production specification, and it enables the sensor to check for its functional reliability during running applications, or to check a change of sensitivity, due to outer pollution, aging and fouling of the membrane.

According to Table 4 that shows the regression results between the primary line-sensor signals $a_{1}^{I}$ and $a_{1}^{II}$, the experiments demonstrate a highly correlated response behavior of both line-sensors with Pearson’s squared correlation coefficients at $R^{2}=0.9999$. The line-sensors show nearly the same slopes (regression coefficient $c_{1}$ in Table 4) with deviations smaller than 1%. The distance of the response of line-sensors 1 and 2 (intercept $c_{0}$ in Table 4) increases with increasing concentration of the internal standard. If $c_{1}=1$, which according to Table 4 is a good approximation, then the difference $a_{1}^{I}-a_{1}^{II}$ will be independent of the outer concentration, which can be considered as an ideal scaling behavior. This difference can be calculated based on the intercept $c_{0}$, i.e., for a discrete point near $\Delta \chi_{CO2}^{i}=\Delta \chi_{CO2}^{a}$. A comparison of $1/c_{0}=1/a_{1}^{I}(\Delta \chi_{CO2}^{i}=\Delta \chi_{CO2}^{a})$ with $d\alpha /da_{1d}$ from the investigated complete concentration range shows a deviation smaller than 0.4%. Thus, also a simple regression of the primary line-sensor responses can be used to estimate the inner slope with $k^{i}=\Delta \chi_{CO2}^{i}/c_{0}$. In addition, such a regression can be performed during a running application.

### 4.6. Impact of Fluctuations of Internal Standard Concentration

Section 4.1, Section 4.2, Section 4.3, Section 4.4 and Section 4.5 have essentially considered the behavior of the combined line-sensor. However, as shown in Figure 6, fluctuation of the internal standard concentration influences the dispersion of its response. Such fluctuations, produced during the mixing process by the MFCs, will be recorded as slightly smoothed by the NDIR reference sensor that was situated in the purge gas line between the MFCs and line-sensor 2 (Figure 2). They will be further smoothed on the way to the line-sensor and, in addition, as a result of the comparatively slow diffusion process through the sensor membrane. If so, a smoothing of the NDIR reference sensor signal should result in a more appropriate internal standard signal.

The highest fluctuations of $CO_{2}^{i}$ (Figure 6) were observed in experiment 2. Therefore, the impact of such a smoothing will be considered for this experiment. Taking into account that only older $CO_{2}^{i}$ readings contribute to a combined line-sensor response at a time $t_{k}$, unidirectional moving averages ${\left(l+1\right)}^{-1}\sum_{l}CO_{2}^{i}(t_{k-l})$ were applied over different numbers of readings from l=0 (i.e., no smoothed original data) to 10 to smooth the internal standard concentration at $t_{k}$.

Using the smoothed records of internal standard concentration the respective responses of the combined line-sensor were recalculated according to Equations (5) and (6) for the various concentration plateaus, and mean standard deviations $\delta CO_{2}^{a}$ were determined based on the concentration-weighted standard deviations of this plateau concentrations.

**Figure 7:**
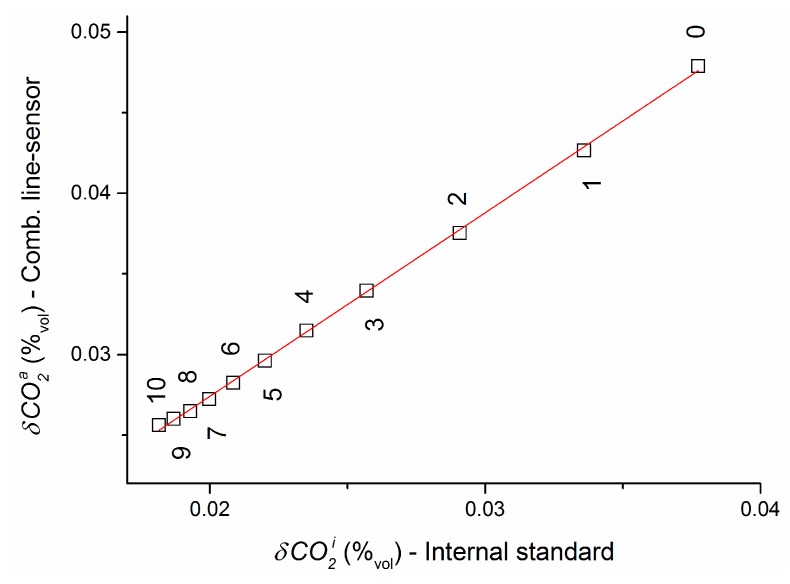
Dispersion $\delta CO_{2}^{a}$ of the combined line-sensor response for experiment 2 in dependence of the dispersion $\delta CO_{2}^{i}$ of internal standard concentration $CO_{2}^{i}$. The underlying original $CO_{2}^{i}$ readings are smoothed using moving averages over l+1 data point (l indicated).

Figure 7 shows the influence of smoothing resulting in a decrease of fluctuations of the internal standard concentration (standard deviation $\delta CO_{2}^{i}$) for increasing size l of the moving average. At the same time, the mean standard deviation $\delta CO_{2}^{a}$ decreases asymptotically. For a size l=10 of moving average, the outer dispersion $\delta CO_{2}^{a}=0.026$%_vol_ of the combined line-sensor response has the same order of magnitude as the equivalent weighted mean dispersion 0.046%_vol_ achieved by the NDIR reference sensor. Linear extrapolation of this smoothing behavior to a (mathematical) constant internal standard concentration results in an expectation value for the asymptotic mean outer dispersion of $\delta CO_{2}^{a}=(0.0046\pm 0.0002)$%_vol_ for the combined line-sensor response. Thus, the most accurate measurement values could be expected for using, e.g., a (certified) gas mixture from a simple gas container.

### 4.7. Measurement Comparison of Combined Line-Sensor and NDIR Reference

**Figure 8:**
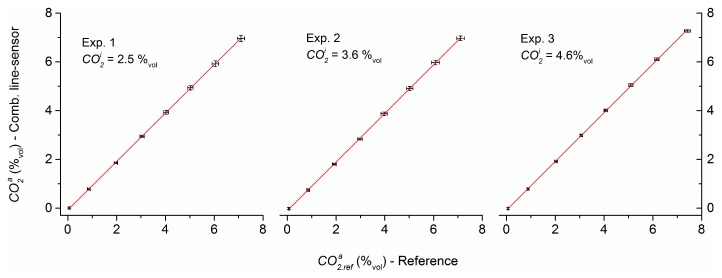
Comparison the of non-calibrated combined line-sensor response with that of the NDIR reference for the concentrations C_1_–C_7_ (error bars show the three-fold standard deviations).

Figure 8 shows as black error bars the result of measurement comparison analyzed with the combined line-sensor $\left(CO_{2}^{a}\pm 3\;\delta CO_{2}^{a}\right)$ and the NDIR reference $\left(CO_{2,ref}^{a}\pm 3\;\delta CO_{2,ref}^{a}\right)$ in the test column for experiments 1–3. These combined line-sensor responses were not calibrated. The smoothing interval of the internal standard concentration was set to l=10. To obtain the dynamic pressure change $a_{1d}$ according to Equation (5), the offset $a_{1s,calc}=-0.0241$ mbar/s (calculated in Section 4.3) was uniformly reduced from $a_{1}^{I}$ in all data records. Linear regressions $CO_{2}^{a}=(b_{1}\pm \delta b_{1})\cdot CO_{2,ref}^{a}+(b_{0}\pm \delta b_{0})$ with the NDIR reference concentration $CO_{2,ref}^{a}$ ($b_{1},\;b_{0}$—regression coefficient and intercept, $\delta b_{1}$, $\delta b_{0}$—respective standard errors) demonstrate the linear behavior of sensor response. The strong similarity of both sensor responses is also documented by the fit constants in Table 5.

For comparison, using the experimentally determined individual offsets $a_{1s,\exp}$ (Section 4.3) instead of the calculated offset $a_{1s,calc}$ in regressions $CO_{2}^{a}=(b_{1,\exp}\pm \delta b_{1,\exp})\cdot CO_{2,ref}^{a}+(b_{0,\exp}\pm \delta b_{0,\exp})$ does not change the regression coefficients $b_{1,\exp}$. It only slightly reduces the distances of the intercepts $b_{0,\exp}$ of regression (Table 6) with respect to the expectation (0.038%_vol_).

The high-level consistency of the sensor responses during the measurement comparison was not a priori expected. Differences are possible in the permeabilities of the PDMS tubes with respect to the PDMS material that was used in [34] for permeability characterization, and the values of permeability can be dependent on the characterization method. In addition, the permeabilities of the C-Flex reference membranes were not considered in this study, tolerances can occur of geometrical measures, and setup-based limitations exist for the exact estimation, e.g., of purge gas pressure. Therefore, further investigations are necessary to obtain a consistent picture about the accuracy of the calibration-free approach, especially, in terms of a reduction of uncertainties in the membrane parameters.

## 5. Summary and Conclusions

Linear membrane-based gas sensors (line-sensors) could be usefully applied in large heterogeneous systems, e.g., for the representative determination of CO_2_ in the subsurface, where they could replace a high number of sensors with local detection. Such line-sensors can be designed as a function of properties of the observation object using membranes of different lengths (range 1–100 m). In addition, the membrane thickness and material can be adapted to the required time resolution, target gas, gas sensitivity and selectivity or environmental conditions. However, besides the operating conditions, the line-sensor response depends on the given gas matrix, the individual site-adapted sensor geometry, the membrane material, and the target gas component. Therefore, a disadvantage is that calibration is needed, especially of the slope, which could change over several orders of magnitude (Section 4.1). Moreover, such (repetitive) calibrations could require much effort, especially in the case of, e.g., large line-sensor lengths.

An approach based on an internal standard has been developed to overcome such a multi-criteria slope dependency. This results in the normalization of line-sensor response. The approach is discussed using the example of CO_2_ measurement in dry air with tubular PDMS membranes for a CO_2_ concentration range of up to 7%_vol_ and various internal standard concentrations of CO_2_.

For a detailed examination two identical single line-sensors were coupled according to Figure 2 to a combined line-sensor and the pressure changes $a_{1}^{I}$ and $a_{1}^{II}$ were analyzed against different gas-tight reference chambers (tubes). To optimize the construction of such a combined line-sensor, these two reference tubes can be substituted by a single reference tube, which will be studied in further investigations. Finally, the selective tubes and such a common reference tube can be integrated, e.g., in a suitable meshwork for mechanical protection and better handling as shown in [30,32].

The inner signal dynamic (inner slope $k^{i}$) that forms the theoretical/measurement-technical background information for sensor configuration, and the influence of variations of temperature and internal standard concentration on the measurement signal, are considered. Comparable measurement results were achieved, irrespective of the concentration of the internal standard that was actually used. However, fluctuations of this concentration propagate into the measurement result and increase its dispersion. Moreover, it could be shown for an 18 K temperature range (278–296 K) that the line-sensor response was not significantly influenced by temperature variations in the sensor’s environment.

The inner slope $k^{i}$ that is the reciprocal value of inner sensitivity of the combined line-sensor can be calculated based on material parameters and used to configure an application specific optimal design of the combined line-sensor. Moreover, based on the measurement values, this parameter can be recorded, e.g., in terms of a technical production specification, and it enables the sensor to check for its functional reliability during running applications. Thereby, a change of $k^{i}$ can be used to monitor an alteration of membrane, e.g., by aging or fouling, and to signal a pollution or clogging of sensor surface. Thus, the sensor detects a measurement value and assesses simultaneously the significance of measurement. Further studies are necessary for an explicit consideration of such an intelligent measurement concept.

By means of a measurement comparison, it was shown that the approach enables accurate gas analysis without any calibration of the combined line-sensor response, resulting in an unexpectedly low bias (deviation in slope <1%) with respect to the NDIR reference sensor GMP-221. Minimization of a theoretically motivated offset of the response function can be performed based on theory, depending on the actual measurement conditions. Such an offset reduction could be implemented in the internal sensor logic.

A comparison of calculated offsets with experimentally determined offsets show a strong dependency of the calculation on the material data, i.e., the permeabilities from the literature. For further enhancement of accuracy, such permeabilities must be analyzed in the relevant (partial) pressure range for the materials of the membrane sets that are actually used.

## Figures and Tables

**Table 1:** Measurement techniques for CO_2_ detection (examples from the literature).

Type of CO_2_ Sensor	Detection/Measurement Range	Operating Temperature (°C)	Remarks	Reference
Solid electrolyte (Nasicon with Na + Ba—based mixed carbonate electrodes)	6 ppm–100%_vol_	~600	High chemical stability, fast response, improved performance against moisture	[12]
Metal oxide (LaOCl and SnO_2_)	400–2000 ppm	350–550	Low cost, high sensitivity, long-term stability; limited accuracy	[13]
Polymer-based	300 ppm–1.5%_vol_	Room temperature	Operational for room temperature and high humidity atmospheres	[14]
Non-dispersive infrared (NDIR)	<10%_vol_	-	Low cost, wide measurement range, accuracy: ±30 ppm/5% typically (may vary with range), characteristic curve producible but may suffer from thermal drift, light scattering effects etc.	[15,16]
Fluorescence based fiber optic	<100%_vol_	-	Chemically inert, sensitivity depends on various support matrices, low cross-sensitive to other gas components	[17,18]
Gas chromatography (atmospheric trace gases, air quality)	50 ppm–100%_vol_	Standard temperature & Pressure (STP) conditions	Static field/laboratory analytical method, high cost, high sensitivity and selectivity, miniaturization potential yet to be explored. Precision: ±0.06 ppm to ±1.29 ppm	[19,20,21,22]

**Table 2:** Experimental scenarios $(CO_{2}^{a},\;CO_{2}^{i}$ (%_vol_)—outer and inner concentration of CO_2_, *T*—temperature, Q—volumetric gas flow).

Exp. No.	Test Gas		Purge Gas	
	Conditions	*T* (K)	Internal Standard $CO_{2}^{i}$ (%_vol_)	*T* (K)
1	$CO_{2}^{a}$ up to 7%_vol_ *Q* = 1.5 L/min	290.5–294.8	≈2.5	291.8–295.7
2		292.3–295.6	≈3.6	293.3–296.4
3		282.1–296.2	≈4.6	283.8–296.9
4		278.0–296.3	≈4.6	277.0–297.3

**Table 3:** Means $\Delta CO_{2}^{i}$ and standard deviations $\delta (\Delta CO_{2}^{i})$ of the internal standard concentrations in experiments 1–3, coefficients $d\alpha /da_{1d}$ and standard errors $\delta (d\alpha /da_{1d})$ of regressions in Figure 5 and calculated inner slopes $k^{i}$ ($\delta k^{i}$—standard deviation).

Exp. No.	$\Delta CO_{2}^{i}\pm \delta (\Delta CO_{2}^{i})$ (%_vol_)	$d\alpha /da_{1d}\pm \delta (d\alpha /da_{1d})$ (s/mbar)	$k^{i}\pm \delta k^{i}$ (s/mbar)
1	2.485 ± 0.026	4.1270 ± 0.0134	0.1047 ± 0.0013
2	3.582 ± 0.036	2.9178 ± 0.0079	0.1040 ± 0.0013
3	4.561 ± 0.024	2.3237 ± 0.0067	0.1055 ± 0.0009

**Table 4:** Regressions of $a_{1}^{I}=(c_{1}\pm \delta c_{1})\cdot a_{1}^{II}+(c_{0}\pm \delta c_{0})$, $c_{1}$ is the slope, $c_{0}$ the intercept, $\delta c_{1}$, $\delta c_{0}$ are the respective standard errors and ε is the standard error of fit.

Exp. No.	$c_{1}\pm \delta c_{1}$	$c_{0}\pm \delta c_{0}$ (mbar/s)	ε
1	1.0016 ± 0.0003	0.2369 ± 0.0001	0.0020
2	0.9924 ± 0.0004	0.3440 ± 0.0001	0.0023
3	0.9921 ± 0.0004	0.4313 ± 0.0001	0.0023

**Table 5:** Results of measurement comparison using the calculated offset $a_{1s,calc}$ ($R^{2}$—Pearson’s squared correlation coefficient, ε—standard error of fit).

Exp. No.	$b_{1}\pm \delta b_{1}$	$b_{0}\pm \delta b_{1}$ (%_vol_)	$R^{2}$	ε
1	0.9922 ± 0.0007	−0.0225 ± 0.0028	0.9996	0.043
2	0.9969 ± 0.0007	−0.0566 ± 0.0029	0.9996	0.045
3	0.9999 ± 0.0007	−0.0335 ± 0.0028	0.9997	0.043

**Table 6:** Results of measurement comparison using the experimentally determined offsets $a_{1s,\exp}$ ($R^{2}$—Pearson’s squared correlation coefficient, ε—standard error of fit).

Exp. No.	$b_{1,\exp}\pm \delta b_{1,\exp}$	$b_{0,\exp}\pm \delta b_{0,\exp}$ (%_vol_)	$R^{2}$	ε
1	0.9921 ± 0.0007	−0.0295 ± 0.0028	0.9996	0.043
2	0.9970 ± 0.0007	−0.0402 ± 0.0029	0.9996	0.045
3	0.9999 ± 0.0007	−0.0283 ± 0.0028	0.9997	0.043

## Appendix A

**Table A1:** Time lag between the responses of line-sensor 1 and the NDIR-reference, mean concentrations $CO_{2}^{i,a}$ and standard deviations $\delta CO_{2}^{i,a}$ for experiments 1–4.

No. of Exp.	Time Lag (s)	Internal Standard Concentrations (%_vol_)		Test-Column Concentrations (%_vol_)		
		$CO_{2}^{i}$	$\delta CO_{2}^{i}$	Name	$CO_{2}^{a}$	$\delta CO_{2}^{a}$
1	805	2.523	0.026	C_0_	0.059	0.009
				C_1_	0.859	0.019
				C_2_	1.968	0.023
				C_3_	3.045	0.029
				C_4_	4.027	0.032
				C_5_	5.025	0.037
				C_6_	6.045	0.042
				C_7_	7.096	0.050
2	630	3.618	0.036	C_0_	0.061	0.008
				C_1_	0.855	0.020
				C_2_	1.929	0.027
				C_3_	2.975	0.033
				C_4_	3.970	0.043
				C_5_	5.021	0.044
				C_6_	6.077	0.056
				C_7_	7.109	0.053
3	767	4.597	0.024	C_0_	0.065	0.006
				C_1_	0.882	0.012
				C_2_	2.024	0.019
				C_3_	3.064	0.017
				C_4_	4.064	0.024
				C_5_	5.097	0.030
				C_6_	6.159	0.031
				C_7_	7.420	0.042
4	595	4.598	0.047	C_0_	0.051	0.008
				C_1_	0.845	0.017
				C_2_	2.045	0.039
				C_3_	3.060	0.038
				C_4_	4.004	0.040
				C_5_	4.976	0.053
				C_6_	6.011	0.052
				C_7_	7.031	0.045
